# Pulsed Field Ablation and Neurocardiology: Inert to Efferents or Delayed Destruction?

**DOI:** 10.31083/j.rcm2503106

**Published:** 2024-03-14

**Authors:** Derek Chang, Andrew Arbogast, Ikeotunye Royal Chinyere

**Affiliations:** 1 Banner University Medicine, Banner Health, Tucson, AZ 85719, USA; 2 Sarver Heart Center, University of Arizona, Tucson, AZ 85724, USA

**Keywords:** irreversible electroporation, cardiac, neurons, autonomic nervous system, ganglia, plexi, nervous system

## Abstract

**Background:**

The therapeutic use of irreversible electroporation in clinical cardiac laboratories, termed pulsed field ablation (PFA), is gaining pre-regulatory approval momentum among rhythm specialists for the mitigation of arrhythmogenic substrate without increased procedural risk. Though electroporation has been utilized in other branches of science and medicine for decades, apprehension regarding all the possible off-target complications of PFA have yet to be thoroughly identified and investigated.

**Methods:**

This brief review will summarize the preclinical and adult clinical data published to date on PFA’s effects on the autonomic system that interplays closely with the cardiovascular system, termed the neurocardiovascular system. These data are contrasted with the findings of efferent destruction secondary to thermal cardiac ablation modalities, namely radiofrequency energy and liquid nitrogen-based cryoablation.

**Results:**

*In vitro* neurocardiology findings, *in vivo* neurocardiology findings, and clinical neurocardiology findings to date nearly unanimously support the preservation of a critical mass of perineural structures and extracellular matrices to allow for long-term nervous regeneration in both cardiac and non-cardiac settings.

**Conclusions:**

Limited histopathologic data exist for neurocardiovascular outcomes post-PFA. Neuron damage is not only theoretically possible, but has been observed with irreversible electroporation, however regeneration is almost always concomitantly described.

## 1. Introduction

In the modern era of catheter-based arrhythmogenic substrate suppression, thermal modalities have long held the evidence-based guideline recommendations for both atrial [1] and ventricular [2] abnormalities. As such, these temperature-based technologies predominate in clinical electrophysiology labs and operating rooms across the world. The use of cryoballoon ablation for atrial fibrillation (AF) management was found to be non-inferior to radiofrequency (RF) ablation, with a comparable, if not improved, procedural workflow, safety profile, and complication rate [3]. Nonetheless, multiple serious but infrequent adverse events have marked these thermal techniques, including esophageal perforation [4], phrenic nerve injury or palsy [5,6], pulmonary vein stenosis [7], and increased risk of thromboembolic events [8]. In addition, the durability of thermal ablation for AF has been found to wane rather quickly for single procedures, with approximately 60% of patients back in AF after approximately two years [9].

Irreversible electroporation (IRE), the technique underlying pulsed field ablation (PFA), has broad clinical applications beyond cardiology, particularly within oncology, with DNA/RNA translocation into cells for anti-tumor effects [10], delivering chemotherapeutics into tumor cells [11], and notably ablating solid tumors near nervous structures without permanent damage [12,13]. Current data on the clinical use of catheter-based PFA for arrhythmia management, though limited, support PFA as a method with increased myocardial tissue specificity, with the main advantage of minimal ancillary damage, and non-inferior short-term success rates [14,15].

PFA is being considered as an alternative therapy option in the armamentarium of rhythm specialists that could tune to precisely target arrhythmogenic foci in either the atria or ventricles [16] with minimal collateral damage to surrounding structures such as the nervous tissue (example: phrenic nerve) or esophagus, as can be observed with thermal ablation techniques. Despite these exciting applications, there are still safety concerns with PFA procedures, specifically regarding potential neurocardiac [17] disruptions. High-risk mediastinal structures in the cardiac nervous system include efferent axons from the stellate ganglion and sympathetic ganglia, which facilitate sympathetic tone, cardiac-specific axons from the tenth cranial nerve (vagus), which facilitate parasympathetic outputs, as well as cervical and vertebral ganglia axons from the superficial and/or deep cardiac plexi [18-20]. Equally complex in distribution and number are the afferent neurons responsible for returning information to the central nervous system (example: parasympathetic nodose ganglia), as well as visceral interneurons that contribute to the intrinsic cardiac nervous system. These structures form a highly complex and likely functionally redundant network that allow for precise control of cardiac function via subconscious cortico-brainstem reflexes in conjunction with autonomic outputs. Disruption of this neurocardiovascular system via neuron lysis/necrosis or delayed apoptosis has been documented secondary to thermal cardiac ablation or surgical procedures, however preservation may be feasible with a nonthermal ablation technique that affords sufficient specificity to cardiomyocytes. This succinct review aims to high-light neurological outcomes in preclinical and clinical cardiac ablation procedures, with an emphasis on the epicardial efferent axons and ganglia.

## 2. *In Vitro* Neurocardiology Findings

Early *in vitro* studies determined that clusters of ganglia derived from the intrinsic cardiac autonomic system (ANS), known as ganglionated plexi (GP), were critically involved in the initiation and sustenance of reentrant tachyarrhythmias [21,22]. Interest has been growing regarding the IRE parameters needed to selectively ablate cardiomyocytes while preserving neuronal function, to potentially reduce adverse ablation events. The IRE threshold of 375 V/cm has been described as the minimum for irreversibly ablating cardiomyocytes [23], though specifically in reference to immature rodent myoblasts, which may not be representative of mature human cardiomyocytes. However, the exact threshold for selective GP ablation remained elusive.

In more recent studies, it was found that increasing the total number of pulses, regardless of field threshold at either 1000 V/cm or 1250 V/cm produced a greater proportion of delayed (24 hours post-electroporation) cardiomyocyte death [24]. Interestingly, at various field strengths ranging from 12.5 to 1250 V/cm, neuronal cell lines were found to be similarly susceptible to cell death when compared to cardiomyocytes [24]. When adjusted for biphasic pulses with longer intra-pulse intervals (increased interphase delay), the extent of cell death, for both cardiac and neural lines, was increased due to a minimization of any cancellation effect [25]. Though these *in vitro* data suggest GPs could be targeted with cardiac IRE protocols, other studies have suggested that phrenic nerves require exposure to much higher field strengths to facilitate sustained cell death [26,27]. This may be partly due to neurons having cell bodies proximal to the site of ablation, inherently having larger cell body diameters, and being myelinated, providing electrical insulation.

## 3. *In Vivo* Neurocardiology Findings

In the context of GP ablation isolation, GPs are inextricably tied to the central nervous system (CNS) and ANS, which likely potentiate pathologic cardiac fibrosis and tachyarrhythmia susceptibility. This is illustrated in patients who have undergone cardiac allotransplantation and exhibit a low permanent AF incidence, possibly due to cardiac denervation secondary to mediastinal manipulation.

Preclinical investigation with canine models demonstrated that PFA to epicardial GPs effectively reduced AF inducibility post-ablation with no signs of collateral nervous damage [28]. IRE performed on sciatic nerves in adult rats, yielded a pattern of post-IRE myelin disintegration, followed by Wallerian degeneration and finally regeneration of axons with no quantifiable functional deficits seven weeks post-procedure, compared to sham control rodents [29]. Interestingly, the field strength utilized in this rodent study approximated 3800 V/cm, 10-fold above the *in vitro* cardiomyocyte threshold previously described [23]. IRE performed on rabbit sciatic nerve implicated in a solid tumor revealed similar findings in which the nerve axons disintegrated, and then later had signs of nerve repair with improved functional improvement in the rabbits’ ability to stand with near-normal toe-spreading reflexes [30].

These findings have been replicated in additional rabbit studies [31], as well as large animal models [32,33]. Identified porcine studies utilized a gradation of IRE field strengths ranging from 1000 to 2500 V/cm (Table 1, Ref. [29,30,32,34-36]). Under histopathology, the external architecture of the axons was frequently preserved post-IRE, whereas RF produced extensive axonal, extracellular matrix, and collagen denaturation not conducive to regeneration. Although lesions created with IRE did exhibit histopathological signs of nerve damage, the preserved external architecture allowed the regeneration of the axons [32].

## 4. Clinical Neurocardiology Findings

Though the number of clinical studies aimed at describing neuronal functional deficits secondary to PFA are increasing [37], not one study designed to detect damage specific to the neurocardiovascular system could be identified. Only one study appropriately powered to provide molecular insights, in addition to companion physiologic data, into the non-cardiac neurologic effects of cardiac PFA was found.

In a small (n = 18) single-arm prospective observational study [34], patients suffering from symptomatic paroxysmal AF underwent pulmonary vein isolation via PFA to assess therapeutic response and residual cardiac nerve function. Using serologic nerve injury biomarkers (neurofilament light chain, glial fibrillary acidic protein, brain-type fatty acid binding protein, S100*β*) as surrogates of structural neurologic damage, there were no significant differences pre-versus immediately post-ablation or 24 hours post-ablation. Further, the calculated heart rate variability exemplified high specificity to cardiac tissues without signs of collateral nerve damage in the neighboring structures. This data is corroborated by the lack of phrenic nerve paresis or palsy during PFA pulsing with no primary neurological events after short-term follow up.

In a larger (n = 91) prospective cohort study, S100*β* was found to increase (*p* < 0.001) in the blood twenty-one minutes after pulmonary vein isolation with PFA, however the increase was less than that observed twenty minutes after pulmonary vein isolation with cryoballoon ablation [38]. When S100*β* was normalized to the concomitant high-sensitivity troponin-I, PFA produced a ratio approximately four times smaller than cryoballoon’s ratio, suggesting less relative neurotoxcitiy.

## 5. Autonomic Effects of Atrial PFA for AF

Increases in heart rate post-thermal pulmonary vein isolation for AF have been documented up to 2 months post-ablation in patients [39]. This thermal ablation-heart rate finding was confirmed in two repeat clinical studies [38,40], and is contrasted with no significant change in heart rate over 1-month post-PFA [34] or 3 months post-PFA [40]. It was postulated that this effect was due to retained GP and/or a lesser degree of cytotoxicity to the local neurocardiac system. Interestingly, in a single study based out of Germany, approximately half of the PFA pulmonary vein isolation patients exhibited transient bradycardia [41], however this was without any serologic evidence of increased neurocardiac damage relative to a thermal study arm.

## 6. Conclusions

Catheter-based pulsed field ablation is a promising tool for invasive electrophysiologists to manage arrhythmogenic substrates. With comparable procedural skill requirements, a strong safety profile, and decent antiarrhythmic effect durability, one of the few remaining challenges for widespread PFA adoption is possible neurologic consequences. This review emphasized the effects of PFA on the neurocardiovascular system, but due to the limited data, also included data from somatic and cranial nerve studies as well. The data reveal that PFA is capable of affecting neuronal structures and inducing damage, however the preservation of perineural structures allows for long-term regeneration with minimal innervation deficits. These data are not supported by appropriately rigorous clinical studies that incorporate molecular parameters in addition to physiologic data and histopathology, given that no such studies exist to date.

## Figures and Tables

**Table 1.** Summary of pulsed field ablation studies targeting neurologic sequelae.

Reference	Aim(s)	Study design	Study population	Results	Summary/implications
Li W *et al.* [29] (2011). “The Effects of IRE on Nerves”. PLoS ONE 6(4): e18831.	Observing neurological changes post-IRE ablation in rat sciatic nerves to study the long-term effects on the nerve	*In-vivo* experimental study IRE parameters: 3800 V/cm, direct current (below the upper bound of thermal damage), 10 pulses, each 100 μs long	Rat sciatic nerves	- 3 days after IRE, the measured sciatic functional index (SFI), nerve conduction velocity (NCV), and proximal compound muscle action potential (CMAP) was significantly decreased compared to control (without IRE) - 1 week after IRE, disintegrated myelin sheaths and basal membranes were present on histology - Muscles innervated by the sciatic nerve returned to normal function starting 7-weeks after IRE - 7–10 weeks after IRE, differences from the control were the same with myelin sheath structures restored to pre-IRE control condition	- Electrophysiological, histological, and functional results show that nerves can attain full recovery after IRE ablation, which is better than bridging nerve gaps with autografting - Nerve regeneration post-IRE follows typical Wallerian degeneration and regeneration of axons - Although not implicated in humans, these results could be extrapolated to nerve resiliency and retaining endoneurial architecture under IRE
Luo X *et al.* [30] (2017). “The Safety of IRE on Nerves Adjacent to Treated Tumors”. World Neurosurg. 2017 Dec; 108: 642–649.	Evaluate safety of IRE on sciatic nerve after ablation in rabbits with implanted tumors superimposed on the nerve	*In-vivo* experimental study IRE Parameters: 1500 V/cm, 70 μs pulses, 90 pulses per ablation	Rabbit sciatic nerves	- Note: Modified Tarlov Score was used to measure functional improvement by ability to stand with toe reflex - Sciatic nerve function was damaged in a short time, but gradually returned to normal after 14 days - Tumor response (assessed by imaging and gross pathology according to size and necrosis liquefaction) was better post-IRE compared to control (tumor implanted without treatment)	- IRE is an effective method of targeting tumors for destruction while preserving surrounding tissues, including blood vessels and nerves - IRE is effective in tissues with a high density of cell wall structures, but less so in those with a high concentration of collagenous and elastic fibers (i.e., preserves vital extracellular matrix for nerve tissue regeneration) - IRE can cause axonal swelling, disintegration, axon loss, and fibrous tissue hyperplasia 7 days after IRE. At day 28, there are signs of nerve repair, Schwann cell proliferation, and regeneration of proximal axons towards the basement membrane
Schoellnast H *et al.* [32] (2011). “Acute and Subacute Effects of IRE on Nerves: Experimental Study in a Pig Model”. Radiology 260(2): 421–427.	Evaluate whether IRE has the potential to damage porcine models and compare to histopathologic findings after RFA	*In-vivo* experimental study IRE parameters: 1350–2250 V (voltage was chosen to keep a constant voltage per distance in tissue of 1500 V/cm), electrode exposure 2 cm, electrode spacing 0.9–1.5 cm, 90 pulses of alternating polarity, pulse length 70 μsec	Porcine sciatic nerves	- Note: Ability to stand up without assistance on the treated limb was used to measure functional improvement - 4 out of 5 animals were able to stand up after 3 days post-procedure (last animal was euthanized) - The IRE lesions created a well-demarcated focus at the middle gluteal muscle, which resulted in nerve thickening, perineural edema, axonal swelling, Schwann cell hyperplasia, and distal Wallerian degeneration - Increased S100 immunostaining that indicates proliferation of Schwann cells and fibroblast proliferation	- There was histopathological evidence of nerve damage after IRE (axonal swelling, axonal fragmentation, Wallerian degeneration), but external architecture was not affected with Schwann cell hyperplasia taking place 6–14 days after - In contrast, RFA produced extensive collagen denaturation of the external architecture, which is a sign of thermal damage and less a bility for nerve regeneration - IRE has the potential to damage nerves but retains the external architecture for regeneration
Guo F *et al.* [34] (2023). “Effects of pulsed field ablation on autonomic nervous system in paroxysmal atrial fibrillation: A pilot study”. Heart Rhythm 20(3): 329–338.	Assess PFA system to (1) achieve acute PVI; (2) impact on cardiac ANS function and levels of nerve injury markers; (3) assess cerebral microemboli by DWI-MRI	Pilot Clinical Study Single-arm prospective, observational study IRE Parameters: 1800 V, alternating current, biphasic, micro-second length	Symptomatic paroxysmal AF patients (n = 18) scheduled to receive PFA	- Nerve injury biomarkers (NfL, GFAP, B-FABP, and S100β) showed no significant differences in levels pre-ablation, immediate post-ablation, or 24 hours after - Calculated cardiac vagal and sympathetic tones via HRV showed no significant differences in HR pre-ablation and 30-days post-ablation - No phrenic nerve paresis or palsy captured during PFA pulses to the right superior PV - Kaplan-Meier estimates of freedom from recurrent AF were 83 ± 9% with no primary neurological adverse events 8 months post-ablation	- Using nerve injury biomarkers (known to be elevated after cryoablation with high predictive value in assessing neurocognitive damage, post-stroke, CV surgery, and cardiac arrest), frequency and time domain indices of HRV, and follow-up data, showed high specificity to cardiac tissues using PFA while showing no evidence of collateral damage to nerve tissue around ablation site, including the cardiac nerve plexus - Limitations: small sample size, single site, did not compare PFA vs. radiofrequency ablation
Ellis TL *et al.* [35] (2011). “Nonthermal IRE for intracranial surgical applications”. Journal of Neurosurgery 114(3): 681–688.	Assess the novel nonthermal irreversible electroporation (NTIRE) approach in selectively ablating brain tissue without collateral damage for intracranial surgery	*In-vivo* experimental study IRE Parameters: 500–2000 V, 9 sets of 10 alternating polarity pulses (50 μsec each, 3.5 seconds in total), 4 Hz	Canine brains	- No significant deterioration in neurological ability or coma scale scores from baseline evaluations with an absence in seizure events - Intraoperative ultrasound showed clearly demarcated hypoechoic zone with hyperechoic rim within targeted brain parenchyma - MRI performed immediately post-op showed fluid accumulation within ablation sites and focal disruption of the blood-brain barrier - 48-hour post-NTIRE MRI studies showed edema in the subcortical white matter adjacent to the zone of ablation without objective clinical effects - Histopathological sections showed NTIRE lesions have a submillimeter line of demarcation between areas of necrosis and normal brain tissue	- NTIRE showed that pulsed IRE can cause targeted cell death without disrupting extracellular matrix, axons, and major blood vessels in adjacent non-CNS tissues - Specific electric field parameters need to be defined on specific tissue types - Tissue heterogeneity (gray vs. white matter) are important to define as brain cancer tissue most likely requires different electric field parameters compared to normal tissue - Although there was concern for vasogenic edema, no clinical or neurological deterioration was observed
Schoellnast H *et al.* [36] (2013). “The delayed effects of IRE ablation on nerves”. European Radiology 23(2): 375–380.	Evaluate the delayed effects of IRE (2-month follow-up from reference [32])	*In-vivo* experimental study IRE Parameters: 1650–2100 V (voltage was chosen in order to keep a constant voltage per distance in tissue, 1500 V/cm), electrode exposure 2 cm, electrode spacing 1.1–1.4 cm, 90 pulses of alternating polarity, pulse length of 70 μs	Porcine sciatic nerves	- No clinical signs of “lameness” after 4 weeks post-IRE - Proximal CMAP dropped within a range of 21–97% (>50% was considered significant) with half of the animals (3/6) severely affected clinically - No anatomic gross changes were observed post-mortem - After 2 months, axonal regeneration, Schwann cell hyperplasia, ellipsoids (collapsed myelin), and perineurial fibrosis was present on histopathology in 75–100% of the neural fascicles expressing S100 for all subjects	- Large number of small caliber axons expressing neurofilaments (closely associated with hyperplastic Schwann cells consistent with axon regeneration), indicates that regenerated axons were smaller than normal axons of untreated control nerves - Follow-up nerve conduction studies after 2 months showed diminished CMAP amplitude, which may indicate limited functional recovery. It is possible that larger nerve fascicles in different animal models may be more resilient. However, clinical observation showed full recovery, which may describe compensation by the other muscles (e.g., gluteus, thigh extensors/adductors). - Limitation: follow-up time-period might be too short to truly describe the full capacity of the nerve to regenerate

IRE, irreversible electroporation; V/cm, volts per centimeter; RFA, radiofrequency ablation; PFA, pulsed field ablation; PVI, pulmonary vein isolation; ANS, autonomic nervous system; DWI, diffusion-weighted imaging; MRI, magnetic resonance imaging; AF, atrial fibrillation; NfL, neurofilament light chain; GFAP, glial fibrillary acidic protein; B-FABP, brain-type fatty acid binding protein; S100*β*, calcium-binding protein B beta; HRV, heart rate variation; PV, pulmonary vein; CV, cardiovascular; CNS, central nervous system; CMAP, compound muscle action potential.

## References

[1] Arbelo E, Dagres N. The 2020 ESC atrial fibrillation guidelines for atrial fibrillation catheter ablation, CABANA, and EAST. Europace: European Pacing, Arrhythmias, and Cardiac Electrophysiology: Journal of the Working Groups on Cardiac Pacing, Arrhythmias, and Cardiac Cellular Electrophysiology of the European Society of Cardiology. 2022; 24: ii3–ii7. 35661865 10.1093/europace/euab332

[2] Könemann H, Dagres N, Merino JL, Sticherling C, Zeppenfeld K, Tfelt-Hansen J, Spotlight on the 2022 ESC guideline management of ventricular arrhythmias and prevention of sudden cardiac death: 10 novel key aspects. Europace: European Pacing, Arrhythmias, and Cardiac Electrophysiology: Journal of the Working Groups on Cardiac Pacing, Arrhythmias, and Cardiac Cellular Electrophysiology of the European Society of Cardiology. 2023; 25: euad091. 37102266 10.1093/europace/euad091 PMC10228619

[3] Wörmann J, Lüker J, van den Bruck JH, Filipovic K, Erlhöfer S, Scheurlen C, Pulmonary vein isolation for atrial fibrillation using true high-power short-duration vs. cryoballoon ablation. Clinical Research in Cardiology: Official Journal of the German Cardiac Society. 2023; 112: 846–852. 37009942 10.1007/s00392-023-02188-2 PMC10241727

[4] Ha FJ, Han HC, Sanders P, Teh AW, O’Donnell D, Farouque O, Prevalence and prevention of oesophageal injury during atrial fibrillation ablation: a systematic review and meta-analysis. Europace: European Pacing, Arrhythmias, and Cardiac Electrophysiology: Journal of the Working Groups on Cardiac Pacing, Arrhythmias, and Cardiac Cellular Electrophysiology of the European Society of Cardiology. 2019; 21: 80–90. 29912306 10.1093/europace/euy121

[5] Heeger CH, Sohns C, Pott A, Metzner A, Inaba O, Straube F, Phrenic Nerve Injury During Cryoballoon-Based Pulmonary Vein Isolation: Results of the Worldwide YETI Registry. Circulation. Arrhythmia and Electrophysiology 2022; 15: e010516. 34962134 10.1161/CIRCEP.121.010516 PMC8772436

[6] Sacher F, Monahan KH, Thomas SP, Davidson N, Adragao P, Sanders P, Phrenic nerve injury after atrial fibrillation catheter ablation: characterization and outcome in a multicenter study. Journal of the American College of Cardiology. 2006; 47: 2498–2503. 16781380 10.1016/j.jacc.2006.02.050

[7] Saad EB, Rossillo A, Saad CP, Martin DO, Bhargava M, Erciyes D, Pulmonary vein stenosis after radiofrequency ablation of atrial fibrillation: functional characterization, evolution, and influence of the ablation strategy. Circulation. 2003; 108: 3102–3107. 14623799 10.1161/01.CIR.0000104569.96907.7F

[8] Yokoyama K, Nakagawa H, Wittkampf FHM, Pitha JV, Lazzara R, Jackman WM. Comparison of electrode cooling between internal and open irrigation in radiofrequency ablation lesion depth and incidence of thrombus and steam pop. Circulation. 2006; 113: 11–19. 16380552 10.1161/CIRCULATIONAHA.105.540062

[9] Luik A, Kunzmann K, Hörmann P, Schmidt K, Radzewitz A, Bramlage P, Cryoballoon vs. open irrigated radiofrequency ablation for paroxysmal atrial fibrillation: long-term FreezeAF outcomes. BMC Cardiovascular Disorders. 2017; 17: 135. 28545407 10.1186/s12872-017-0566-6 PMC5445510

[10] Daud AI, DeConti RC, Andrews S, Urbas P, Riker AI, Sondak VK, Phase I trial of interleukin-12 plasmid electroporation in patients with metastatic melanoma. Journal of Clinical Oncology: Official Journal of the American Society of Clinical Oncology. 2008; 26: 5896–5903. 19029422 10.1200/JCO.2007.15.6794 PMC2645111

[11] Martin CH, Martin RCG. Optimal Dosing and Patient Selection for Electrochemotherapy in Solid Abdominal Organ and Bone Tumors. Bioengineering (Basel, Switzerland). 2023; 10: 975. 37627860 10.3390/bioengineering10080975 PMC10451240

[12] Yarmush ML, Golberg A, Serša G, Kotnik T, Miklavčič D. Electroporation-based technologies for medicine: principles, applications, and challenges. Annual Review of Biomedical Engineering. 2014; 16: 295–320. 10.1146/annurev-bioeng-071813-104622 24905876

[13] Davalos RV, Mir ILM, Rubinsky B. Tissue ablation with irreversible electroporation. Annals of Biomedical Engineering. 2005; 33: 223–231. 15771276 10.1007/s10439-005-8981-8

[14] Reddy VY, Koruth J, Jais P, Petru J, Timko F, Skalsky I, Ablation of Atrial Fibrillation with Pulsed Electric Fields: An Ultra-Rapid, Tissue-Selective Modality for Cardiac Ablation. JACC. Clinical Electrophysiology 2018; 4: 987–995. 30139499 10.1016/j.jacep.2018.04.005

[15] Turagam MK, Neuzil P, Schmidt B, Reichlin T, Neven K, Metzner A, Safety and Effectiveness of Pulsed Field Ablation to Treat Atrial Fibrillation: One-Year Outcomes From the MANIFEST-PF Registry. Circulation. 2023; 148: 35–46. 37199171 10.1161/CIRCULATIONAHA.123.064959

[16] Repp ML, Chinyere IR. Opportunities and Challenges in Catheter-Based Irreversible Electroporation for Ventricular Tachycardia. Pathophysiology: the Official Journal of the International Society for Pathophysiology. 2024; 31: 32–43. 38251047 10.3390/pathophysiology31010003 PMC10801500

[17] Aksu T, Gupta D, Pauza DH. Anatomy and Physiology of Intrinsic Cardiac Autonomic Nervous System: Da Vinci Anatomy Card #2. JACC. Case Reports 2021; 3: 625–629. 34317590 10.1016/j.jaccas.2021.02.018 PMC8302792

[18] Shivkumar K, Ajijola OA, Anand I, Armour JA, Chen PS, Esler M, Clinical neurocardiology defining the value of neuroscience-based cardiovascular therapeutics. The Journal of Physiology. 2016; 594: 3911–3954. 27114333 10.1113/JP271870 PMC4945719

[19] Dusi V, Zhu C, Ajijola OA. Neuromodulation Approaches for Cardiac Arrhythmias: Recent Advances. Current Cardiology Reports. 2019; 21: 32. 30887264 10.1007/s11886-019-1120-1 PMC7105505

[20] Sridharan A, Bradfield JS, Shivkumar K, Ajijola OA. Autonomic nervous system and arrhythmias in structural heart disease. Autonomic Neuroscience: Basic & Clinical. 2022; 243: 103037. 36201902 10.1016/j.autneu.2022.103037

[21] Scherlag BJ, Yamanashi W, Patel U, Lazzara R, Jackman WM. Autonomically induced conversion of pulmonary vein focal firing into atrial fibrillation. Journal of the American College of Cardiology. 2005; 45: 1878–1886. 15936622 10.1016/j.jacc.2005.01.057

[22] O’Brien B, Reilly J, Coffey K, González-Suárez A, Quinlan L, van Zyl M. Cardioneuroablation Using Epicardial Pulsed Field Ablation for the Treatment of Atrial Fibrillation. Journal of Cardiovascular Development and Disease. 2023; 10: 238. 37367403 10.3390/jcdd10060238 PMC10299113

[23] Kaminska I, Kotulska M, Stecka A, Saczko J, Drag-Zalesinska M, Wysocka T, Electroporation-induced changes in normal immature rat myoblasts (H9C2). General Physiology and Biophysics. 2012; 31: 19–25. 22447827 10.4149/gpb_2012_003

[24] Avazzadeh S, O’Brien B, Coffey K, O’Halloran M, Keane D, Quinlan LR. Establishing Irreversible Electroporation Electric Field Potential Threshold in A Suspension In Vitro Model for Cardiac and Neuronal Cells. Journal of Clinical Medicine. 2021; 10: 5443. 34830725 10.3390/jcm10225443 PMC8622402

[25] Vižintin A, Vidmar J, Ščančar J, Miklavčič D. Effect of interphase and interpulse delay in high-frequency irreversible electroporation pulses on cell survival, membrane permeabilization and electrode material release. Bioelectrochemistry (Amsterdam, Netherlands). 2020; 134: 107523. 32272337 10.1016/j.bioelechem.2020.107523

[26] Hunter DW, Kostecki G, Fish JM, Jensen JA, Tandri H. In Vitro Cell Selectivity of Reversible and Irreversible: Electroporation in Cardiac Tissue. Circulation. Arrhythmia and Electrophysiology 2021; 14: e008817. 33729827 10.1161/CIRCEP.120.008817

[27] Mercadal B, Arena CB, Davalos RV, Ivorra A. Avoiding nerve stimulation in irreversible electroporation: a numerical modeling study. Physics in Medicine and Biology. 2017; 62: 8060–8079. 28901954 10.1088/1361-6560/aa8c53 PMC5744675

[28] Madhavan M, Venkatachalam KL, Swale MJ, Desimone CV, Gard JJ, Johnson SB, Novel Percutaneous Epicardial Autonomic Modulation in the Canine for Atrial Fibrillation: Results of an Efficacy and Safety Study. Pacing and Clinical Electrophysiology: PACE. 2016; 39: 407–417. 26854009 10.1111/pace.12824 PMC4846530

[29] Li W, Fan Q, Ji Z, Qiu X, Li Z. The effects of irreversible electroporation (IRE) on nerves. PloS One. 2011; 6: e18831. 21533143 10.1371/journal.pone.0018831 PMC3077412

[30] Luo X, Qin Z, Tao H, Shi J, Fang G, Li Z, The Safety of Irreversible Electroporation on Nerves Adjacent to Treated Tumors. World Neurosurgery. 2017; 108: 642–649. 28927918 10.1016/j.wneu.2017.09.049

[31] Kwon JH, Kim MD, Kim SH, Lee EW, Kahlid SA. Effects of irreversible electroporation on femoral nerves in a rabbit model. Minimally Invasive Therapy & Allied Technologies: MITAT: Official Journal of the Society for Minimally Invasive Therapy. 2022; 31: 306–312. 32744129 10.1080/13645706.2020.1799820

[32] Schoellnast H, Monette S, Ezell PC, Deodhar A, Maybody M, Erinjeri JP, Acute and subacute effects of irreversible electroporation on nerves: experimental study in a pig model. Radiology. 2011; 260: 421–427. 21642418 10.1148/radiol.11103505 PMC6939978

[33] Wong SSM, Hui JWY, Chan AWH, Chu CM, Rowlands DK, Yu SCH. Irreversible Electroporation of the Femoral Neurovascular Bundle: Imaging and Histologic Evaluation in a Swine Model. Journal of Vascular and Interventional Radiology: JVIR. 2015; 26: 1212–1220.e1. 26071841 10.1016/j.jvir.2015.04.023

[34] Guo F, Wang J, Deng Q, Feng H, Xie M, Zhou Z, Effects of pulsed field ablation on autonomic nervous system in paroxysmal atrial fibrillation: A pilot study. Heart Rhythm. 2023; 20: 329–338. 36435350 10.1016/j.hrthm.2022.11.013

[35] Ellis TL, Garcia PA, Rossmeisl JH Jr, Henao-Guerrero N, Robertson J, Davalos RV. Nonthermal irreversible electroporation for intracranial surgical applications. Laboratory investigation. Journal of Neurosurgery. 2011; 114: 681–688. 20560725 10.3171/2010.5.JNS091448

[36] Schoellnast H, Monette S, Ezell PC, Maybody M, Erinjeri JP, Stubblefield MD, The delayed effects of irreversible electroporation ablation on nerves. European Radiology. 2013; 23: 375–380. 23011210 10.1007/s00330-012-2610-3 PMC9377791

[37] Ekanem E, Reddy VY, Schmidt B, Reichlin T, Neven K, Metzner A, Multi-national survey on the methods, efficacy, and safety on the post-approval clinical use of pulsed field ablation (MANIFEST-PF). Europace: European Pacing, Arrhythmias, and Cardiac Electrophysiology: Journal of the Working Groups on Cardiac Pacing, Arrhythmias, and Cardiac Cellular Electrophysiology of the European Society of Cardiology. 2022; 24: 1256–1266. 35647644 10.1093/europace/euac050 PMC9435639

[38] Lemoine MD, Mencke C, Nies M, Obergassel J, Scherschel K, Wieboldt H, Pulmonary Vein Isolation by Pulsed-field Ablation Induces Less Neurocardiac Damage Than Cryoballoon Ablation. Circulation. Arrhythmia and Electrophysiology 2023; 16: e011598. 36938715 10.1161/CIRCEP.122.011598

[39] Tang LYW, Hawkins NM, Ho K, Tam R, Deyell MW, Made L, Autonomic Alterations After Pulmonary Vein Isolation in the CIRCA-DOSE (Cryoballoon vs Irrigated Radiofrequency Catheter Ablation) Study. Journal of the American Heart Association. 2021; 10: e018610. 33634706 10.1161/JAHA.120.018610 PMC8174287

[40] Musikantow DR, Neuzil P, Petru J, Koruth JS, Kralovec S, Miller MA, Pulsed Field Ablation to Treat Atrial Fibrillation: Autonomic Nervous System Effects. JACC. Clinical Electrophysiology 2023; 9: 481–493. 36752473 10.1016/j.jacep.2022.10.028

[41] Tohoku S, Schmidt B, Schaack D, Bordignon S, Hirokami J, Chen S, Impact of Pulsed-Field Ablation on Intrinsic Cardiac Autonomic Nervous System After Pulmonary Vein Isolation. JACC. Clinical Electrophysiology 2023; 9: 1864–1875. 37480870 10.1016/j.jacep.2023.05.035

